# Childhood Adversities and Caregiving for Older Parents: Building Capacity for a Caring Society

**DOI:** 10.1093/geronb/gbae083

**Published:** 2024-05-14

**Authors:** Bo Hu, Xue Bai, Pengyun Wang

**Affiliations:** Care Policy and Evaluation Centre, Department of Health Policy, London School of Economics and Political Science, London, UK; Institute of Active Ageing, Department of Applied Social Sciences, The Hong Kong Polytechnic University, Hong Kong, China; Department of International Trade, School of Economics, Nankai University, Tianjin, China; Oxford Internet Institute, University of Oxford, Oxford, UK; (Social Sciences Section)

**Keywords:** Caregiving for parents, Caring society, Childhood adversities, Labor shortage, Projection model

## Abstract

**Objectives:**

This study investigates the relationships between childhood adversities and the provision of informal care for older parents in later life in China.

**Methods:**

The data came from 4 waves of the China Health and Retirement Longitudinal Study (*N* = 20,047). Using multilevel logistic regression models, we examined the relationships between adverse experiences in childhood and both the propensity and intensity of caregiving for older parents. Drawing on the regression results, we then estimated the total number of caregivers for older parents in China.

**Results:**

Experiencing 1 additional childhood adversity was associated with a decrease of 8% in the odds of providing informal care (*p* < .001). The association between childhood adversity and caregiving remained significant after sociodemographic factors and later-life outcomes were controlled for. We estimated that 58.3 million middle-aged adults in China were providing care for parents in 2020. Had people experienced 1 fewer adversity in their childhood, there would have been 2.2 million more caregivers in 2020. Had they experienced 2 fewer adversities, there would have been 3.4 million more caregivers.

**Discussion:**

The factors associated with informal caregiving can be traced back to early-life experiences. To address the shortage of informal care supply, it is crucial to foster a caring culture from the very beginning of human development.

Informal care provided by family members is crucial to the well-being of older people who require support to perform daily activities such as eating, dressing, and cooking. In most countries, care responsibilities are primarily undertaken by informal caregivers. Providing adequate support and high-quality care is essential for mitigating the progression of functional disability among older people and enabling them to stay longer in their homes (Wilcox et al., 1994). By contrast, insufficient care is associated with various life consequences for older people, such as unmet care needs, early admission to care homes, poor mental health, and high mortality (Gaugler et al., 2005; He et al., 2015; Hu & Chou, 2022).

The past few decades have witnessed a decrease in the proportion of younger adults in many parts of the world, and this trend is expected to continue in the decades to come (World Health Organization, 2015). In China, for example, the number of younger adults aged between 45 and 64 years old is projected to decrease by 21%, from 408 million in 2020 to 321 million in 2060, whereas the number of older adults aged 65 years or older is projected to rise by 139%, from 179 million in 2020 to 428 million in 2060 (United Nations, 2022). Adult children play an indispensable role in providing informal care and serve as a primary source of support for their older parents (Coe & Van Houtven, 2009). Indeed, the majority of caregivers for older parents are younger adults aged between 45 and 64 years old (Pickard, 2015). The challenges associated with population aging not only include a rapid increase in the demand for care but also loom large in anticipation of a shortage of younger caregivers.

To address the issue of caregiver shortage, it is paramount to thoroughly understand the factors associated with the informal care supply (Broese van Groenou & De Boer, 2016; Schmidt et al., 2016). However, most of the existing studies have focused on the contemporary determinants of caregiving. An important but overlooked issue is early-life experience. There has been much debate in the literature about the role played by a person’s care attitude, health conditions, and socioeconomic circumstances, but it should be recognized that these are also life outcomes that are continually shaped and reshaped by experience throughout the life course and profoundly affected by major events in earlier stages of life. Exposure to childhood adversities such as maltreatment, family separation, and household dysfunction can have long-standing impacts on a person’s life outcomes, which in turn affects the decisions to provide care for family members. The understanding of informal caregiving remains incomplete without an in-depth study of the influence of early-life experiences.

In this study, we investigated the relationship between childhood adversities and caregiving for older parents in China, and on this basis estimated the total number of caregivers for older parents. We argue that taking a life course perspective in the analyses opens up new avenues for appraising the drivers of informal care supply, prompting researchers and policymakers to re-think the policy measures that can be implemented to tackle the caregiver shortage and build a caring society.

## Literature Review

### Factors Associated With Informal Caregiving

Much research has been done in the scientific community to understand the factors associated with informal caregiving for family members. Broese van Groenou & De Boer (2016) developed the Informal Care Model. According to this model, the provision of informal care is driven by the disposition of caregivers, the needs of care recipients, and contextual factors (e.g., support from social networks and related services). The disposition of caregivers can be further categorized into three types of motivations, namely attitudes toward care (“Do I want to?”), sense of obligation (“Do I have to?”), and barriers to and facilitators of caregiving (“Can I?”). In parallel, Schmidt et al. (2016) maintained that whether potential caregivers decide to provide care for family members is dependent on the overall amount and types of resources that they possess. The theory specified that material, health, and caring resources are crucial to the provision of informal care.

The Informal Care Model and the resource-based model originate from different theoretical backgrounds and differ in terms of how they conceptually link various groups of factors. However, they resonate with each other considerably with regard to implications for empirical research. Socioeconomic status is discussed from the perspective of material resources in the resource-based model but analyzed as a facilitating/prohibiting factor in the Informal Care Model (i.e., the “Can I?” factor). People with a low socioeconomic status have to spend great efforts and energy to bring sufficient financial resources to the household and make ends meet, which leaves them with less time to provide care (Brandt et al., 2009). In comparison, people with higher socioeconomic status are more integrated into their family networks, which strengthens their intergenerational solidarity and propensity to provide mutual support within their families (Coimbra et al., 2013). An extensive body of evidence shows that people with higher levels of income and education are more likely to become informal caregivers (Baji et al., 2019; de Klerk et al., 2021; Schmidt et al., 2016; Wang et al., 2021). Meanwhile, negative association has also been observed in studies focusing on particular subgroups of caregivers such as those with intensive caregiving responsibilities (Kelle, 2020) or caring for people with severe care needs (Tokunaga & Hashimoto, 2017).

The health status of caregivers reflects their intrinsic capability to provide care for their family members. Intrinsic capability is treated as a health resource in the resource-based model and a facilitator/prohibitor of caregiving in the Informal Care Model. The “healthy caregiver hypothesis” posits that people with better health have a higher propensity to provide care, whereas poor health is a major obstacle to becoming a caregiver (Bertrand et al., 2012). McCann et al. (2004), Fredman et al. (2009), and Schmidt et al. (2016) showed that people with higher levels of physical functioning are more likely to become caregivers or to continue providing care. Bertrand et al. (2012) reported that caregivers exhibited significantly more favorable cognitive performance than noncaregivers.

In addition to health and economic resources, the Informal Care Model emphasizes the key role of normative beliefs as an independent driving force of informal caregiving (Broese van Groenou & De Boer, 2016). The Model indicates that, in specific social settings, caregivers feel “obliged” or feel that they are “expected” to provide care to varying degrees (i.e., the “Do I have to?” factor). This argument is particularly relevant in the context of China, where cultural traditions and practical norms stress the responsibility of middle-aged adults to provide care for their older parents. This cultural expectation is rooted in traditional Chinese family values, such as filial piety and intergenerational solidarity, whereas the practical norms in relation to caregiving are due to a lack of well-funded public pensions, especially for the large rural population of China (Shi & Hu, 2020).

### Childhood Adversities and Later-Life Outcomes

Life course research points to a fundamental issue in human development: later-life outcomes do not take shape in 1 day; instead, many of them can be linked back to people’s experiences in childhood and are shaped and reshaped by events throughout the life course (McEwen & McEwen, 2017). Childhood is a critical stage of human development where intensive psychological and biological programming takes place. Traumatic events or toxic stressors experienced in childhood can have negative impacts on children’s cognitive development and mental and physical health, which tend to persist until adulthood or older age (Ben-Shlomo & Kuh, 2002; Hertzman et al., 2001; Lund et al., 2008). Poor health outcomes can interact with an individual’s psychological, social, and economic circumstances, which leads to a proliferation of adversities. Poor cognitive performance and mental health caused by childhood adversities prevent children from fully benefiting from the education system. This leaves them in a more disadvantaged position later on in terms of educational attainment and financial security. Disadvantaged socioeconomic status, in return, has negative impacts on people’s health (Foverskov et al., 2020; Ihle et al., 2018). It is through this adversity proliferation process that cumulative advantage/disadvantage emerges, inequalities are amplified, and life outcomes are clustered in the population (Dannefer, 2020; Pearlin, 2010).

Childhood experiences also influence attitudes toward caregiving in later life (i.e., the “Do I want to” factor). The attachment theory maintains that childhood adversities compromise children’s attachment system, leading to insecure attachment and poor social functioning in adulthood (Thomson & Jaque, 2017). Attachment security refers to the sense of confidence displayed by an individual that others will be responsive and supportive when needed. The development of the attachment system is substantially influenced by the availability of responsive and supportive caregivers in childhood who are often children’s parents (Ainsworth et al., 2015; Bowlby, 1997) and exhibit a certain degree of continuity after they reach adulthood (Fraley et al., 2011). A number of studies have investigated the relationships between attachment security and planned/actual caregiving for older parents in adulthood. Cicirelli (1983, 1993) showed that secure attachment is associated with more hours of care and less stress attributable to caregiving for older parents. Carpenter (2001) reported that fearful insecurity style is associated with adult daughters providing less care for their mothers and feeling a higher level of stress. In addition, adult children with secure attachment are found to be more prepared and more willing to provide care for older parents (Karantzas et al., 2010; Sörensen et al., 2002).

It should be stressed that people do not experience childhood adversities in a vacuum. Some adverse experiences emerge in connection with historical and social contexts. For example, families in the United States were deeply affected by the Great Depression in terms of family dynamics, coping strategies, and parenting styles, which shaped children’s lifelong attitudes toward family relations (Elder, 1999). Children experiencing the 1959–1961 Chinese famine went through prolonged malnutrition in childhood and had poorer cognitive functioning (Xu et al., 2018) and physical health (Huang et al., 2010) in adulthood. People in successive birth cohorts may be exposed to different social and economic events that have unique impacts on their early-life experience and exert profound influences on their later-life outcomes (Elder et al., 2003).

## Hypothesis

The existing literature suggests that, given a social or historical context, adverse experiences in childhood are associated with deprivation of health and material resources and a less positive attitude toward caregiving in adulthood. These disposition factors or life outcomes in turn affect behavioral patterns of caregiving for family members. Based on these two strands of literature, our central hypothesis posits that adults experiencing childhood adversities provide less care for their older parents or parents-in-law. If this hypothesis is supported by evidence, we expect that reducing the prevalence of childhood adversities will contribute to an increase in the supply of caregivers for older parents in the population.

## Research Methods

### Data and Sample

The data for this study came from four waves of the China Health and Retirement Longitudinal Study (CHARLS). The baseline survey of CHARLS took place in 2011 and collected health and aging-related information from 17,708 individuals aged 45 years old and over in 450 villages and urban communities across China. Follow-up surveys were conducted in 2013, 2015, and 2018, respectively. Refreshed samples were recruited in the follow-up surveys to maintain the representativeness of the entire sample (Zhao et al., 2020). A life history survey was conducted in 2014, which collected information on individuals’ childhood circumstances. All individuals who participated in the CHARLS 2011 and 2013 were invited to participate in the life history, and those who did were followed up in CHARLS 2015 and 2018.

The CHARLS 2015 did not contain information on caregiving for older parents, so was excluded from our analyses. A total of 24,064 individuals participated in the three longitudinal surveys (i.e., CHARLS 2011, 2013, and 2018), among whom 19,170 individuals participated in the life history survey. We further excluded 8,942 individuals who did not have any living parents or parents-in-law across all waves. The rest (10,228 individuals) were the focus of our analyses. The number of those individuals participating in the longitudinal surveys once, twice, and thrice was 3,653, 3,331, and 3,244, respectively, which amounted to a total of 20,047 participants (i.e., observed data points) in our pooled sample.

### Measurements

The outcome variables pertain to the provision of informal care for older parents or parents-in-law. Survey participants were asked whether they “took care of (their) parents or parents-in-law during the last year in assisting them with their daily activities or other activities (e.g., household chores, meal preparation, laundry, going out, grocery shopping, financial management, etc.).” We created a binary variable: 0 = not providing care and 1 = providing care. For those providing care, they were further asked how many hours of care they provided in a week. We then created a binary variable to measure care intensity: 0 = providing less than 10 hr of care (low-intensity care) and 1 = providing more than 10 hr of care (high-intensity care). We chose 10 hr of caregiving as the threshold because this is the point where caregivers start to feel the negative impacts of caregiving on their employment, physical health, and mental well-being (Brimblecombe & Cartagena Farias, 2022; King & Pickard, 2013).

We investigated 13 types of childhood adversities included in the CHARLS life history survey: parental death, parental anxiety, parental depression, parental abnormality of mind, severe disability of a parent, a bedridden parent, no affection, neglect, repeated physical abuse by a parent, parental drug abuse, parental alcohol abuse, parental involvement in criminal activities, and frequently witnessing domestic violence.

We measured childhood adversities in two different ways. First, following previous studies by Schafer and Ferraro (2012) and Brimblecombe et al. (2018), we dichotomized each type of adversity (0 = *no* and 1 = *yes*) and added up the total number of childhood adversities (range: 0–13). Although childhood adversities may take different forms at the societal level, they converge on similar consequences at the biological level. As toxic stressors, they result in the excessive and prolonged activation of the stress-management system in the body, leading to an unbalanced physiological state and compromising the development of children’s brain architecture in the long run (McEwen & McEwen, 2017). The count measure can shed light on the dose-response relationships between child adversities and caregiving for older parents.

Second, we clustered different types of adversities into four broader groups: poor mental health of parents (anxiety, depression, and abnormality of mind), poor physical health of parents (severe disability and bedridden), maltreatment (no affection, neglect, and repeated physical abuse), and dysfunctional household (parental death, alcohol abuse, drug abuse, criminal activity, and domestic violence). This measure appreciates the fact that different types of adversities in childhood may affect later-life outcomes via different pathways and helps to clarify their unique impacts on caregiving propensity and intensity.

We investigated four groups of covariates: demographic variables, family structure, health status, and socioeconomic conditions. We examined three demographic variables including age, gender, and rural–urban residence. There were three variables capturing family structure in our analyses: marital status (0 = *married* and 1 = *never married*, *widowed*, *separated*, or *divorced*), whether or not living with parents or parents-in-law in the same household (0 = *no* and 1 = *yes*), and the total number of living parents or parents-in-law. Individuals who experienced parental death in childhood or whose parents had poor health might have fewer living parents when they reach adulthood, resulting in a lower level of demand for care. Because our focus was on the association between childhood adversities and the care supply, we controlled for the number of living parents/parents-in-law in the analyses.

Regarding health status, we analyzed the participants’ ability to perform activities of daily living (ADLs) or instrumental ADLs (IADLs), cognitive functioning, self-reported health, and depressive symptoms. The CHARLS questionnaire assessed six ADL tasks and five IADL tasks. Each task was measured on a four-point scale. We added up the scores for the ADL (range: 0–18) and IADL tasks (range: 0–15), respectively. A higher score indicates lower functional capability. The cognitive functioning variable came from the harmonized CHARLS data set (Phillips et al., 2021). The variable has a range between 0 and 40, with a higher score indicating poorer cognitive functioning. Self-reported health was measured on a 5-point scale: 1 = *very good* and 5 = *very poor*. Depressive symptoms were measured by the 10-item Center for Epidemiological Studies—Depression (CES-D; Radloff, 1977) Scale. Survey participants were asked to rate eight negative statements and two positive statements on a 4-point scale. We reverse-scored the positive statements and added up the scores of the 10 items. A higher score indicates more severe depressive symptoms (range: 0–30).

We focused on four socioeconomic variables in the analyses: educational achievement, household income per capita, household wealth per capita, and retirement. The education variable has three categories: 1 = *primary education or below*, 2 = *secondary education*, and 3 = *higher education*. The income and wealth variables were logarithmically transformed so that their distributions were approximately symmetric. Retirement is a binary variable: 0 = *still working* and 1 = *formally retired*.

### Statistical Analyses

The statistical analyses consisted of two stages. The first stage focused on testing the key hypothesis of this study. We conducted regression analyses to investigate whether there is a significant association between childhood adversities and caregiving for parents. Health and socioeconomic conditions in later life may mediate the association between childhood adversities and caregiving according to the discussion in the previous sections. We included them as control variables in the regression analyses to elucidate whether childhood adversities have an independent effect on caregiving above and beyond those later-life outcomes. Multilevel regression modeling with random intercepts was constructed to account for intra-individual correlation over time and inter-individual heterogeneity (Rabe-Hesketh & Skrondal, 2012). The proportions of missing values are nontrivial in cognitive impairment, depressive symptoms, income, and wealth. The multiple imputation with chained equations technique was used to utilize all of the information available (van Buuren et al., 2006). The regression analyses were based on five multiply imputed data sets. Stata version 17 was used for the data analysis.

In the second stage, we estimated the total number of caregivers for parents in China. We excluded people aged 70 and over because the proportion of caregivers in this age group is tiny (1.4%). Drawing on the data published by the United Nations (2022), our estimation started with the Chinese population aged between 45 and 69 years old broken down by age and gender in 2020. We then broke down the population into smaller groups according to the number of childhood adversities based on the data reported in the CHARLS. The regression analyses conducted in the first stage enabled us to estimate the predicted probability of caregiving for parents according to age, gender, and the number of childhood adversities. Multiplying the number of people by the predicted probability of caregiving gave us the number of caregivers in each small group. Aggregating the number of caregivers across all groups led to a national estimate of caregivers for parents in 2020. Furthermore, we investigated what the current level of care supply would look like, had people experienced fewer adversities in childhood. We focused on three hypothetical scenarios (1) experiencing one fewer adversity in childhood; (2) experiencing two fewer adversities; and (3) no adversity.

## Results

### Association Between Childhood Adversities and Caregiving

Table 1 shows the characteristics of the pooled sample. Around one-third of the sample (35%) had no experience of any of the 13 childhood adversities, 19% had experienced two adversities, and 17% had experienced three or more childhood adversities. A breakdown of the number and broader types of childhood adversities according to birth cohorts of survey participants is shown in Supplementary Table 1. More than half of the sample (54%) were aged between 45 and 54 years old, and only 8% were aged 65 years old and over (Table 1). An overwhelming majority of the sample (95%) were married, 13% were living with parents/parents-in-law, and around half (52%) had only one living parent/parent-in-law. Additionally, 16% of the participants had received higher education, and 10% were formally retired.

**Table 1.** Sample Characteristics (*N* = 20,047)

Variable	Mean	%	Number of participants
Number of adversities (0–13)	1.29		20,047
No adversity		35	6,962
One adversity		29	5,863
Two adversities		19	3,885
Three or more adversities		17	3,337
Age groups			
45–49		27	5,332
50–54		27	5,448
55–59		23	4,633
60–64		15	2,919
65+		8	1,615
Gender			
Women		50	10,067
Men		50	9,961
Rural/urban residence			
Rural areas		70	14,046
Urban areas		30	5,950
Marital status			
Married		95	19,101
Single		5	924
Living with parents/parents-in-law			
No		87	17,492
Yes		13	2,555
Number of living parents/parents-in-law			
One		52	10,461
Two		32	6,325
Three		12	2,375
Four		4	886
ADL scores (0–18)	0.28		19,973
IADL scores (0–15)	0.51		19,945
Self-reported health (1–5)	2.88		19,477
Cognitive impairment (0–0)	19.63		15,259
Depressive symptoms (1–30)	7.87		18,704
Education			
Primary education		34	6,825
Secondary education		50	9,972
Higher education		16	3,224
Household income per capita	9.29		11,328
Household wealth per capita	10.36		12,850
Retired			
No		90	17,784
Yes		10	1,984

*Note*: ADL = activities of daily living; IADL = instrumental activities of daily living.

Table 2 shows the pooled sample according to their caregiving status, the number of childhood adversities, and broader types of adversities. Among those participants not providing any care, 34% had experienced no childhood adversities, 20% had experienced two childhood adversities, and 17% had experienced three or more adversities. On average they reported 1.31 adversities. Among those providing more than 10 hr of care, 39% reported experiencing no childhood adversities, 16% had experienced two adversities, and 14% had experienced three or more childhood adversities. The average number of adversities was 1.15. These differences are statistically significant. Among participants not providing care, 24% reported that their parents had poor mental health, 20% reported that their parents had poor physical health, 36% reported maltreatment by their parents in childhood, and 21% grew up in a dysfunctional household. Among those providing more than 10 hr of care, the proportions of participants reporting these four broader types of adversities were 21%, 18%, 33%, and 17%, respectively. These differences are statistically significant.

**Table 2.** Bivariate Analysis of Childhood Adversities and Caregiving for Parents (*N* = 20,047)

Childhood adversities	No care (80%, *n* = 15,960)		<10 hr (8%, *n* = 1,657)		>10 hr (12%, *n* = 2,430)		χ^2^ or *F*
	%	Mean	%	Mean	%	Mean	
Total number of adversities							
No childhood adversity	34		36		39		
One adversity	29		30		30		
Two adversities	20		19		16		
Three or more adversities	17		16		14		χ^2^ (6) = 40.6***
Total number of adversities		1.31		1.26		1.15	*F*(2) = 16.29***
Types of adversities							
Poor mental health of parents	24		23		21		
Poor physical health of parents	20		18		18		χ^2^ (2) = 6.7*
Maltreatment	36		36		33		χ^2^ (2) = 10.1**
Household dysfunction	21		20		17		χ^2^ (2) = 23.1***

*Notes*: Poor mental health of parents: anxiety, depression, and abnormality of mind; poor physical health of parents: severe disability and bedridden; maltreatment: no affection, neglect, and repeated physical abuse; dysfunctional household: parental death, alcohol abuse, drug abuse, criminal activity, and domestic violence.

*p < .05. **p < .01. ***p < .001.

The association between the number of childhood adversities and caregiving propensity was statistically significant in a multilevel regression model (Table 3). Experiencing one additional childhood adversity was associated with a decrease of 8% in the odds of providing care for older parents (*p* < .001, Column 2). As we added the three groups of control variables in sequence to the regression analyses, the odds ratios gradually increased to 0.943, 0.955, and 0.962, respectively (columns 3–5). The odds ratios were increasingly closer to 1 and the level of statistical significance decreased, indicating that the association between childhood adversities and caregiving was partly explained by the control variables. Nonetheless, the odds ratio of childhood adversities remained statistically significant after all of the control variables were considered.

**Table 3.** Association Between Childhood Adversities and Propensity of Caregiving in Adulthood (*N* = 20,047, Multilevel Logistic Regression Models)

Variable	Model 1	Model 2	Model 3	Model 4
Number of adversities	0.923*** (0.015)	0.943*** (0.016)	0.955** (0.016)	0.962* (0.016)
Demographic factors				
50–54 years old		0.937 (0.05)	0.959 (0.052)	0.939 (0.051)
55–59 years old		0.798*** (0.048)	0.824*** (0.05)	0.801*** (0.05)
60–64 years old		0.793*** (0.057)	0.858* (0.063)	0.837* (0.063)
65 years old and over		0.818* (0.074)	0.910 (0.085)	0.873 (0.085)
Men		0.781*** (0.034)	0.755*** (0.034)	0.737*** (0.034)
Living in urban areas		1.288*** (0.058)	1.186*** (0.056)	1.062 (0.055)
Family structure				
Single people		1.537*** (0.146)	1.602*** (0.154)	1.615*** (0.156)
Living with parents		1.950*** (0.111)	1.948*** (0.112)	1.946*** (0.113)
Number of living parents		1.278*** (0.033)	1.264*** (0.033)	1.256*** (0.033)
Health outcomes				
ADL scores			1.019 (0.026)	1.019 (0.026)
IADL scores			0.960* (0.018)	0.962* (0.018)
Self-reported health			0.854** (0.049)	0.851** (0.049)
Cognitive impairment			0.974*** (0.005)	0.980*** (0.005)
Depressive symptoms			0.998 (0.004)	1.019 (0.026)
Socioeconomic conditions				
Secondary education				1.003 (0.055)
Higher education				1.383*** (0.104)
Household income				1.025* (0.011)
Household wealth				0.994 (0.007)
Retired				1.180* (0.091)
Time dummies				
2013		0.796*** (0.039)	0.788*** (0.039)	0.807*** (0.04)
2018		0.938 (0.046)	0.880* (0.045)	0.891* (0.046)

*Notes*: ADL = activities of daily living; IADL = instrumental activities of daily living. Outcome variable: 0 = no caregiving and 1 = providing care for parents or parents-in-law.

Figures inside and outside the parentheses are the odds ratio and standard error, respectively.

*p < .05. **p < .01. ***p < .001; imputed data set with five imputations.

Most of the control variables were significantly associated with caregiving propensity. People in younger age groups and women are more likely to provide care (Table 3). Living with parents/parents-in-law doubled the odds of being a caregiver, and an increase of one living parent/parent-in-law increased the odds of being a caregiver by 26%. Health outcomes and socioeconomic conditions in later-life matter. People with a lower level of functional capability, a higher level of cognitive impairment, and poorer self-reported health had lower odds of providing care. The odds of caregiving were positively associated with the level of education and household income per capita. One additional childhood adversity had a similar effect to an increase in the IADL scale by one unit and had a larger effect than an increase in the cognitive impairment scale by one unit.

Conditional upon providing care for parents/parents-in-law, childhood adversities were associated with the intensity of caregiving (Table 4). An increase in one childhood adversity was associated with a decrease of 7% in the odds of providing more than 10 hr as opposed to less than 10 hr of care per week (*p* < .01, Column 2). The statistically significant association remained after we controlled for confounders and later-life outcomes in the regression model (*p* < .05, Column 5). Among the control variables, family structure variables were most strongly associated with caregiving. Living with parents/parents-in-law increased the odds of providing high-intensity care (i.e., >10 hr in a week) by 3.5 times, and an increase in one living parent or parent-in-law was associated with a rise in the odds of providing high-intensity care by 42%. In addition, people with a higher level of education and a lower level of wealth were more likely to provide high-intensity care. Experiencing two additional childhood adversities had a similar effect to that of completing only primary education, as opposed to finishing secondary education.

**Table 4.** Association Between Childhood Adversities and Intensity of Caregiving (*N* = 4,087, Multilevel Binary Logistic Regression Models)

Variable	Model 1	Model 2	Model 3	Model 4
Number of adversities	0.927** (0.027)	0.942* (0.029)	0.928* (0.030)	0.934* (0.031)
Demographic factors				
50–54 years old		1.161 (0.116)	1.123 (0.118)	1.114 (0.119)
55–59 years old		1.178 (0.132)	1.150 (0.136)	1.158 (0.14)
60–64 years old		1.347* (0.183)	1.324 (0.191)	1.355* (0.204)
65 years old and over		1.463* (0.253)	1.400 (0.256)	1.434 (0.273)
Men		0.955 (0.075)	0.995 (0.084)	0.94 (0.083)
Living in urban areas		1.005 (0.084)	1.022 (0.095)	1.015 (0.104)
Family structure				
Single people		1.176 (0.201)	1.211 (0.218)	1.232 (0.225)
Living with parents		3.33*** (0.403)	3.503*** (0.450)	3.562*** (0.464)
Number of living parents		1.392*** (0.071)	1.408*** (0.076)	1.417*** (0.077)
Health outcomes				
ADL scores			0.954 (0.055)	0.961 (0.056)
IADL scores			1.038 (0.042)	1.036 (0.042)
Self-reported health			0.992 (0.113)	0.957 (0.11)
Cognitive impairment			0.993 (0.011)	0.999 (0.012)
Depressive symptoms			1.015 (0.008)	1.016 (0.008)
Socioeconomic conditions				
Secondary education				1.154 (0.126)
Higher education				1.356* (0.199)
Household income				0.997 (0.024)
Household wealth				0.969* (0.014)
Retired				0.984 (0.145)
Time dummies				
2013		0.924 (0.088)	0.931 (0.092)	0.943 (0.094)
2018		0.821* (0.078)	0.822* (0.082)	0.830 (0.084)

*Notes*: ADL = activities of daily living; IADL = instrumental activities of daily living. Outcome variable: 0 = less than 10 hr of caregiving and 1 = more than 10 hr of caregiving.

Figures inside and outside the parentheses are the odds ratio and standard error, respectively.

*p < .05. **p < .01. ***p < .001; imputed data set with five imputations.

We conducted further analyses to check the robustness of our regression results. First, we constructed multilevel-ordered logistic regression models to investigate the relationships between childhood adversities and caregiving (outcome variable: 1 = *no caregiving*, 2 = *less than 10 hr*, and 3 = *more than 10 hr*). Second, to capture the potentially non-linear relationships, we treated the number of childhood adversities as a categorical variable. The dose-response relationships between childhood adversities and caregiving can still be observed (Supplementary Tables 2 and 3). Finally, we investigated the relationships between the broader types of adversities and caregiving in multilevel-ordered logistic regression models. Growing up with parents with poor mental health and experiencing maltreatment in childhood were significantly associated with providing less care for parents in later life (Supplementary Table 4).

Based on the regression results in Model 4 in Tables 3 and 4, we estimated that 58.3 million Chinese people aged between 45 and 69 provided care for parents or parents-in-law in 2020, among whom 23.7 million people provided less than 10 hr and 34.5 million people provided more than 10 hr of care per week. We estimated that there were 28.2 million male caregivers and 30.1 million female caregivers (Column 2, Table 5). In a hypothetical scenario where people had experienced one fewer adversity in their childhood, we estimated there would have been 2.2 million more caregivers in 2020 (i.e., 60.5–58.3 = 2.2, Column 3). Had people experienced two fewer adversities, there would have been 3.4 million more caregivers in 2020 (Column 4). Had people experienced none of the adversities under investigation in this study, the total number of caregivers would have been 62.6 million. This represents an increase of caregivers by 4.4 million people (7.5%; Column 5). It can be noted that the increase in the number of caregivers is more concentrated on people providing high-intensity care (i.e., 38.3–34.5 = 3.8 million) than low-intensity care (i.e., 24.3–23.7 = 0.6 million). This reflects the fact that a reduction in the number of childhood adversities is associated with a higher propensity of caregiving as well as providing more hours of care, as shown in Tables 3 and 4.

**Table 5.** Estimated Number of Caregivers for Parents in China in 2020 (Million People)

Caregivers	National estimates	One fewer adversity	Two fewer adversities	No adversity
Male caregivers	28.2	29.3	29.9	30.4
Female caregivers	30.1	31.2	31.8	32.3
Low-intensity caregivers	23.7	24.1	24.2	24.3
High-intensity caregivers	34.5	36.4	37.5	38.3
Total number of caregivers	58.3	60.5	61.7	62.6
Increase in caregivers	—	2.2	3.4	4.4

*Note*: Figures may not add exactly due to rounding.

## Discussion

This study investigated the associations between adverse childhood experiences and the provision of informal care for older parents in later life. Two-thirds of our sample reported experiencing at least one type of childhood adversity. A high prevalence of childhood adversities has also been reported in other countries. Data collected from 144,017 individuals in 25 states in the United States indicated that 61% of adults had experienced at least one of eight types of childhood adversities (Merrick et al., 2019).

Our central hypothesis was confirmed by the analysis results. Individuals who have experienced a higher number of childhood adversities were less inclined to become caregivers. Moreover, among those who did assume this role, they were less likely to provide high-intensity care for their older parents. People who experienced childhood adversities tended to have fewer living parents (Supplementary Table 5). However, even after we controlled for family structure, health conditions, and socioeconomic status, the association between childhood adversities and caregiving remained statistically significant. It seems that health conditions and socioeconomic status in adulthood mediated the relationship between childhood adversities and caregiver propensity in later life. However, we did not find evidence that those factors mediated the relationship between childhood adversities and caregiving intensity.

The contributions of our study to the existing literature are twofold. First, it extends the analytical lens of informal caregiving to the entire life course. Previous studies have found that informal caregiving is affected by contemporary factors of caregivers such as family structure, health conditions, and socioeconomic status (Baji et al., 2019; Bertrand et al., 2012; de Klerk et al., 2021; Fredman et al., 2009; Schmidt et al., 2016; Wang et al., 2021). Our study shows that, above and beyond those factors, caregiving for older parents is heavily influenced by early-life experiences and events. The direct effects of childhood adversities on caregiving for parents may be attributed to caregiving attitudes, as was highlighted in our review of existing literature.

Second, it contributes to the ongoing debate about the life consequences of childhood adversities. There is abundant evidence that childhood adversities are associated with poorer health and economic outcomes in later life (Brimblecombe et al., 2018; Hu, 2021a, 2021b; Schafer & Ferraro, 2012; Suglia et al., 2022). An increasing number of studies have reported that childhood adversities are equally damaging to social functioning in adulthood, which is characterized by poorer relationships with family members, difficulties in maintaining good friends, and unmet care needs (Brown et al., 2008; Ford et al., 2011; Hu & Wei, 2022; Umberson et al., 2014). Our study adds to this body of literature by showing that people experiencing multiple adversities in childhood demonstrate a weakened propensity to take on care responsibilities later on in their life journeys.

We estimated that there were 58.3 million caregivers aged between 45 and 69 years old providing care for parents or parents-in-law in 2020, accounting for 12% of the Chinese population in this age group. To meet the rising demand for care, a higher proportion of younger adults in the population will need to take on care responsibilities and caregivers will need to provide more hours of care for older parents (Hu et al., 2022). However, if caregivers do increase the intensity of caregiving substantially, they are exposed to health risks and financial worries due to the stress (Coe & van Houtven, 2009) and opportunity costs (Carmichael & Charles, 2003) associated with caregiving. This is a dilemma that many potential caregivers will face in the decades to come.

The caregiver dilemma presents a formidable challenge to Chinese society and beyond. Governments across the world should all think carefully about policies that can enhance both the quantity and productivity of the caregiving workforce. We have shown that had people experienced one fewer adversity in their childhood, there would have been 2.2 million more caregivers in 2020 in China. Had they experienced two fewer adversities, there would have been 3.4 million more caregivers. The majority of the additional caregivers would have been high-intensity caregivers. These findings highlight the importance of paying greater attention to people’s early-life experiences as they are directly linked to caregiving propensity and capability in later life. Safeguarding children from toxic adversities and ensuring they grow up in a caring environment are not only vital for personal development, leaving a lasting impact on their socioeconomic and health trajectories, but can also yield substantial benefits by boosting the overall supply of informal care within society as a whole. The caring environment is likely to be passed down from one generation to the next.

Emphasizing the significance of childhood experience is not meant to deny the essential role played by policies targeted at later-life development and outcomes. In fact, our scenario analyses show that there is an upper limit when it comes to enhancing informal care supply solely through early-life interventions. We found that improved physical and cognitive functioning are also strongly associated with the propensity for caregiving in Chinese informal caregivers, which underscores the need for policies to promote population health and offer support for informal caregivers such as formal care or respite care. These policies help to maintain caregivers’ health and enable them to care for their loved ones in the long run. Equally important are anti-poverty measures because they reduce socioeconomic inequality in society, making sure that caregivers can provide the level of care according to their preference without sacrificing their financial security or living standards. It is by combining all these efforts that the government can better address the challenge of the caregiver shortage.

The limitations of this study should be duly acknowledged. First, childhood adversities were reported retrospectively and thus were vulnerable to recollection bias. This is a limitation shared with all other studies investigating childhood adversities retrospectively. The recall bias may also affect self-reported hours of caregiving. Second, this study did not examine the cohort effects on caregiving. Historical events or social contexts might affect people in a particular cohort more than others and profoundly shape their childhood experiences and later-life outcomes. Future studies that disentangle the interface between historical contexts in China, personal experience, and cohort-based developmental trajectories will greatly enrich our understanding of caregiving behavior in later life. Third, since CHARLS did not collect data pertaining to care attitude or attachment security, we were not able to formally test them as the mediators linking childhood adversities to caregiving activities in later life. Instead, we relied on existing theories and published evidence in this research area to interpret our research findings. Finally, due to data availability, the number of living parents was the only demand-side driver of caregiving we investigated in this study. Care demands of older parents may also vary by their age, care needs, and preferences. In our study, we built multilevel models with random effects to account for such unobserved heterogeneity in care demand. However, this is an indirect way to mitigate the data limitations. In future studies, it would be useful to collect data from the caregiver-recipient dyads and investigate directly the impacts of demand-side drivers on caregiving propensity and intensity.

## Conclusion

From a life course perspective, the factors associated with informal caregiving extend beyond the immediate characteristics of caregivers and can be traced back to early-life experiences. The propensity and capability to provide care are dependent on personal resources, which are accumulated over time and formed through past experiences and events. The shortage of informal caregivers is a major concern shared by numerous countries worldwide. To address this problem, government policies should not only focus on population growth and the labor market but also on realizing the potential of a caring society, with an emphasis on reducing barriers to informal caregiving and nurturing positive attitudes toward care. We argue that to cultivate a caring society, it is crucial to build capacity from the outset of human development.

## Supplementary Material

gbae083_suppl_Supplementary_Tables
