# Supplementary material for: Childhood Adversities and Caregiving for Older Parents: Building Capacity for a Caring Society
Source: J Gerontol B Psychol Sci Soc Sci. 2024 May 14;79(7):gbae083. doi: 10.1093/geronb/gbae083 (PMC11184527; doi:10.1093/geronb/gbae083)
Supplement: gbae083_suppl_Supplementary_Tables [file gbae083_suppl_supplementary_tables.docx]

***The Journals of Gerontology, Series B: Psychological Sciences and Social Sciences* Supplementary Material: Hu et al. Childhood adversities and caregiving for older parents: Building capacity for a caring society.**

Supplementary Table 1. Prevalence of childhood adversities broken down by birth cohorts (N=20,047)

| **Childhood adversities** | Birth cohorts | | | | |
| --- | --- | --- | --- | --- | --- |
|  | Before 1939 | 1940s | 1950s | 1960s | 1970s |
| **Number of childhood adversities** | Proportion (%) | | | | |
| No childhood adversity | 37 | 29 | 33 | 36 | 40 |
| One childhood adversity | 35 | 34 | 29 | 29 | 29 |
| Two childhood adversities | 14 | 19 | 20 | 19 | 19 |
| Three or more adversities | 14 | 18 | 18 | 16 | 12 |
| **Type of childhood adversities** | Proportion (%) | | | | |
| Poor mental health of parents | 16 | 23 | 26 | 23 | 21 |
| Poor physical health of parents | 11 | 18 | 20 | 20 | 17 |
| Maltreatment | 30 | 38 | 37 | 35 | 31 |
| Household dysfunction | 36 | 34 | 25 | 23 | 21 |

Notes: Poor mental health of parents: anxiety, depression, and abnormality of mind; poor physical health of parents: severe disability and bedridden; maltreatment: no affection, neglect, and repeated physical abuse; dysfunctional household: parental death, alcohol abuse, drug abuse, criminal activity, and domestic violence

Supplementary Table 2. Association between childhood adversities and caregiving for parents (N=20,047, multilevel ordered logistic regression models)

| **Variable** | Model 1 | Model 2 | Model 3 | Model 4 |
| --- | --- | --- | --- | --- |
| Number of adversities | 0.897*** (0.018) | 0.925*** (0.018) | 0.933*** (0.019) | 0.942** (0.019) |
| **Demographic factors** | |  |  |  |
| 50-54 years old |  | 0.950 (0.050) | 0.966 (0.051) | 0.948 (0.050) |
| 55-59 years old |  | 0.813*** (0.048) | 0.842** (0.050) | 0.822*** (0.050) |
| 60-64 years old |  | 0.823** (0.058) | 0.886 (0.063) | 0.870 (0.064) |
| 65 years old and over |  | 0.853 (0.077) | 0.949 (0.086) | 0.914 (0.087) |
| Male |  | 0.782*** (0.033) | 0.757*** (0.033) | 0.735*** (0.033) |
| Living in urban areas |  | 1.279*** (0.057) | 1.198*** (0.055) | 1.071 (0.054) |
| **Family structure** |  |  |  |  |
| Single people |  | 1.538*** (0.144) | 1.597*** (0.15) | 1.611*** (0.152) |
| Living with parents |  | 2.145*** (0.121) | 2.144*** (0.121) | 2.141*** (0.121) |
| Number of living parents |  | 1.304*** (0.033) | 1.290*** (0.033) | 1.282*** (0.033) |
| **Health outcomes** |  |  |  |  |
| ADL scores |  |  | 1.016 (0.024) | 1.017 (0.025) |
| IADL scores |  |  | 0.954** (0.017) | 0.955** (0.017) |
| Self-reported health |  |  | 0.964 (0.023) | 0.965 (0.023) |
| Cognitive impairment |  |  | 0.974*** (0.005) | 0.982*** (0.005) |
| Depressive symptoms |  |  | 0.998 (0.004) | 0.999 (0.004) |
| **Socioeconomic conditions** |  |  |  |  |
| Secondary education |  |  |  | 1.028 (0.055) |
| Higher education |  |  |  | 1.414*** (0.104) |
| Household income |  |  |  | 1.025* (0.011) |
| Household wealth |  |  |  | 0.993 (0.007) |
| Retired |  |  |  | 1.171* (0.088) |
| 2013 |  | 0.799*** (0.038) | 0.787*** (0.038) | 0.807*** (0.039) |
| 2018 |  | 0.923 (0.045) | 0.884* (0.044) | 0.895* (0.045) |
| N | 20,047 | 20,047 | 4,087 | 4,087 |

Notes: Outcome variable: 1=no caregiving, 2=less than 10 hours of caregiving, 3=more than 10 hours of caregiving; figures inside and outside the parentheses are the odds ratio and standard error, respectively; *p<0.05, **p<0.01, ***p<0.001; imputed dataset with five imputations.

Supplementary Table 3. Association between childhood adversities and caregiving for parents: Robustness checks (multilevel binary logistic regression models)

| **Variable** | Caregivers vs. non-caregivers | | >10 hrs vs. <10 hrs of care | |
| --- | --- | --- | --- | --- |
|  | Odds ratio (standard error) | | Odds ratio (standard error) | |
| **Childhood adversities** |  |  |  |  |
| No childhood adversity | Ref. | Ref. | Ref. | Ref. |
| One adversity | 0.924 (0.050) | 0.989 (0.052) | 0.949 (0.089) | 0.983 (0.097) |
| Two adversities | 0.765***(0.048) | 0.845** (0.052) | 0.760* (0.083) | 0.799 (0.092) |
| Three or more adversities | 0.758***(0.050) | 0.878* (0.058) | 0.823 (0.096) | 0.856 (0.107) |
| **Control variables** | No | Yes | No | Yes |
| **Multilevel model** | Yes | Yes | Yes | Yes |
| N | 20,047 | 20,047 | 4,087 | 4,087 |

Notes: Ref.: reference category; *p<0.05, **p<0.01, ***p<0.001; imputed dataset with five imputation.

Supplementary Table 4. Association between four broader types of childhood adversities and caregiving for older parents (N=20,047, multilevel ordered logistic regression models)

| **Variable** | Model 1 | Model 2 | Model 3 | Model 4 |
| --- | --- | --- | --- | --- |
| **Type of childhood adversities** |  |  |  |  |
| Poor mental health of parents | 0.774*(0.100) | 0.776*(0.099) | 0.807(0.104) | 0.822(0.106) |
| Poor physical health of parents | 0.928(0.052) | 0.961(0.053) | 0.989(0.055) | 0. 991(0.055) |
| Maltreatment | 0.887**(0.040) | 0.885**(0.040) | 0.890**(0.040) | 0.899*(0.040) |
| Dysfunctional household | 0.826***(0.040) | 0.910(0.046) | 0.912(0.047) | 0.931(0.047) |
| **Control variables** |  |  |  |  |
| Demographic variables | No | Yes | Yes | Yes |
| Family structure | No | Yes | Yes | Yes |
| Health factors | No | No | Yes | Yes |
| Socioeconomic conditions | No | No | No | Yes |
| Time dummies | No | Yes | Yes | Yes |
| **Multilevel model** | Yes | Yes | Yes | Yes |

Notes: Outcome variable: 1=no caregiving, 2=less than 10 hours of caregiving, 3=more than 10 hours of caregiving; figures inside and outside the parentheses are the odds ratio and standard error, respectively; *p<0.05, **p<0.01, ***p<0.001; imputed dataset with five imputations.

Supplementary Table 5. Bivariate association between childhood adversities and number of living parents or parents-in-law (N=20,047)

| **[Childhood adversities** | **No living parent** | **One living parent** | | **Two living parents** |  |
| --- | --- | --- | --- | --- | --- |
|  | Proportion (%) | | | | Pearson χ^2^ test for independence |
| Total number of adversities |  |  | |  |  |
| No childhood adversity | 31 | 47 | | 22 | χ^2^ (6) = 152.9*** |
| One adversity | 34 | 47 | | 19 |  |
| Two adversities | 37 | 47 | | 16 |  |
| Three or more adversities | 38 | 49 | | 13 |  |
|  | **No living parent-in-law** | | **At least one living parent-in-law** | |  |
| Total number of adversities | Proportion (%) | | | | Pearson χ^2^ test for independence |
| No childhood adversity | 36 | | 64 | | χ^2^ (3) = 4.8 |
| One adversity | 37 | | 63 | |  |
| Two adversities | 37 | | 63 | |  |
| Three or more adversities | 38 | | 62 | |  |

Note: *p<0.05, **p<0.01, ***p<0.001
